# Gemcitabine-Loaded Albumin Nanoparticle Exerts An Antitumor Effect on Gemcitabine-Resistant Pancreatic Cancer Cells Induced by MDR1 and MRP1 Overexpression in Vitro

**DOI:** 10.3389/fsurg.2022.890412

**Published:** 2022-05-17

**Authors:** Lei Kong, Jiali Du, Jichun Gu, Junyuan Deng, Yujie Guo, Baian Tao, Chen Jin, Deliang Fu, Ji Li

**Affiliations:** Department of Pancreatic Surgery, Huashan Hospital, Fudan University, Shanghai, China

**Keywords:** pancreatic cancer, gemcitabine resistance, albumin nanoparticle, high MDR1 and MRP1 levels, ATP depletion, in vitro study

## Abstract

**Purpose:**

Gemcitabine (GEM) is the first-line chemotherapeutic drug for pancreatic cancer treatment in clinical practice. However, many reasons can reduce the efficacy of GEM, among which the high expression of ATP-binding cassette (ABC) transporters is a significant factor. In this study, we aimed to investigate the antitumor effect of gemcitabine-loaded human serum albumin nanoparticle (GEM-HSA-NP) on GEM-resistant pancreatic cancer cells induced by the high expression of ABC transporters, namely multidrug resistance protein 1/P-gp/ABCB1 (MDR1) and multidrug resistance-associated protein 1/ ABCC1 (MRP1).

**Methods:**

MDR1 and MRP1 were stably overexpressed via lentiviral transduction in the pancreatic cancer cell lines BxPC3 and PANC1. Proliferation inhibition assays, cell cycle arrest and apoptosis analyses were conducted to examine the antitumor effect of GEM-HSA-NP. In addition, intracellular ATP levels were determined to explore the potential mechanisms implicated preliminarily.

**Results:**

When administered to GEM-resistant cancer cells, GEM-HSA-NP displayed its antitumor effect by promoting the inhibition of proliferation, cell cycle arrest, and apoptosis induction. Intracellular ATP depletion, caused by the albumin component of GEM-HSA-NP was proposed to be potentially involved in the modulation of ABC transporter activity.

**Conclusion:**

GEM-HSA-NP can effectively overcome GEM-resistance induced by MDR1 and MRP1 overexpression, which highlights its potential value in a clinical setting.

## Introduction

Pancreatic cancer has the worst prognosis of all the solid malignant tumors affecting humans, with the associated death rate being nearly equal to the incidence (1). Although surgery is the most effective way to treat pancreatic cancer, a high proportion of patients still experience tumor recurrence and metastasis after surgery, and the 5-year survival rate after radical resection remains less than 20% (2–4). Moreover, due to the difficulty of obtaining an early diagnosis of pancreatic cancer, patients with surgical indications account for only 15% (5). Consequently, chemotherapy remains the best treatment option for most patients with pancreatic cancer.

Gemcitabine (2,2-difluoro-2-deoxycytidine, GEM) is recommended as the first-line chemotherapy drug for pancreatic cancer (6). However, due to primary and acquired drug resistance, the clinical use of GEM chemotherapy is only possible in 20%–30% of pancreatic cancer patients (7). Multiple factors are responsible for the resistance of pancreatic cancer cells to the effects of GEM, including the deficiency or decreased activity of human nucleoside transporters (hNTs) (8, 9), the abnormal expression of intracellular metabolic enzymes for GEM (10, 11), and the increased efficiency of drug efflux pumps at the cell membrane (12). Among these reasons, GEM efflux is regarded as a major hindrance to successful chemotherapy (13).

The overexpression of ATP-binding cassette (ABC) transporters in cancer cells is one of the main mechanisms responsible for drug efflux (14). ABC transporters are a large class of membrane protein complexes that comprises 48 members, which are divided into seven subfamilies (labelled through ABC-A to ABC-G), according to their sequence homology (15). By using energy derived from the hydrolysis of ATP to adenosine diphosphate (ADP), ABC transporters are able to mediate multidrug resistance of cancer and cause chemoresistance by transporting drugs across the membrane against a concentration gradient, thus, reducing intracellular drug accumulation (16, 17). Multidrug resistance protein 1/P-gp/ABCB1 (MDR1) and multidrug resistance-associated protein 1/ABCC1 (MRP1) are two popular and important members of the ABC transporter family (18, 19). MDR1 and MRP1 are significantly expressed in varieties of pancreatic cancer cell lines (20). We selected BxPC3 and PANC1 cell lines for the present project because they represent aggressive types of pancreatic cancer and have been widely investigated in previous researches (21). It has been demonstrated that MDR1- and MRP1-overexpressing pancreatic cancer cells are highly resistant to GEM (22). Clinical researches have confirmed that high expression of MDR1 and MRP1 could result in GEM resistance, and that up-regulation of MDR1 and MRP1 in pancreatic tumors could contribute to a generally poor treatment response and a shorter postoperative survival time (23–26). Thus, it is of critical importance to explore new strategies for inhibiting the function of overexpressed MDR1 and MRP1 in pancreatic cancer cells in order to reverse GEM resistance.

The emergence of nanotechnology provides a new strategy for cancer treatment, and nanomedicines have shown great potential in the application of alternative treatments for anticancer agents (27, 28). By using nanoparticle albumin-bound technology, our team selected human serum albumin (HSA) to encapsulate GEM and successfully prepared the gemcitabine-loaded human serum albumin nanoparticle (GEM-HSA-NP) as a novel gemcitabine-delivery agent. The characterization of GEM-HSA-NP was stable and reproducible. GEM-HSA-NP displayed an average size of 150 ± 27 nm, and with a drug-loading efficiency of 10.42% ± 3.5% and an encapsulation efficiency of 82.99% ± 3.5%, furthermore, it exhibited a nearly spherical shape and distributed evenly under transmission electron microscopy (29). HSA was chosen as the drug carrier due to its excellent biocompatibility, non-immunogenicity and non-toxicity. It was previously acknowledged that exogenous proteins absorbed by the cell would be degraded in an ATP-consuming process (30, 31). GEM-HSA-NP is composed of GEM and albumin, thus, theoretically, after GEM-HSA-NP taken into cancer cells, the degradation of the albumin component within the cell would consume large amounts of ATP. Since ABC transporters are ATP-dependent membrane proteins, we speculated that this ATP-consuming albumin degradation process might competitively limit the ATP supply of ABC transporters and thus decrease their activity. Therefore, we postulated that GEM-HSA-NP could be considered as a possible modulator of ABC transporters to overcome GEM resistance caused by increased drug efflux from pancreatic cancer cells.

In the present study, we constructed single-factorial GEM-resistant pancreatic cancer cell lines by overexpressing MDR1 and MRP1 via lentiviral transduction and used them as a model to evaluate the antitumor effect of GEM-HSA-NP. Furthermore, we measured the variation in intracellular ATP levels to confirm our assumption regarding the effect of GEM-HSA-NP on the activity of ABC transporters and preliminarily delineated the potential mechanism responsible for eliminating GEM-resistance.

## Materials and Methods

### Chemicals and Reagents

Gemcitabine (GEM) hydrochloride was obtained from Hansen Pharmaceutical Co., Ltd. (Jiangsu, People’s Republic of China). GEM-HSA-NP was prepared with nanoparticle albumin-bound technology, by which GEM was mixed with HSA in an aqueous solvent and passed under high pressure through a jet to construct drug albumin nanoparticles, as previously reported (29). Normal saline (NS) was obtained from Shanghai Baxter Healthcare Co., Ltd. (Shanghai, People’s Republic of China). Dulbecco’s modified Eagle’s medium (DMEM), fetal bovine serum (FBS), and phosphate-buffered saline (PBS) were purchased from Bo’ao Biological Technology Co., Ltd. (Shanghai, People’s Republic of China). Cell Counting Kit-8 (CCK8) was purchased from Dojindo (Kyushu, Japan). The Annexin V-APC/PI apoptosis kit was purchased from Multi-Sciences (Hangzhou, People’s Republic of China). The ATP assay kit was purchased from Beyotime Institute of Biotechnology (Shanghai, People’s Republic of China).

### Cell Culture

The BxPC3 and PANC1 cell lines were obtained from the Shanghai Branch of the Chinese Academy of Sciences (Shanghai, People’s Republic of China). The use of these cell lines did not require an ethical statement from the Institutional Review Board. The cells were incubated at 37 °C in a humidified atmosphere of 5% CO_2_. Cells were cultured in DMEM supplemented with 10% FBS, 100 U/mL penicillin, and 100 mg/mL streptomycin.

### Lentivirus Construction and Cell Transduction

To upregulate the expression of target genes, full-length cDNA plasmids containing *MDR1* and *MR*P1 were amplified and cloned into a lentivirus-based vector with the following component sequence: CMV-MCS-3FLAG-EF1-copGFP-T2A-puromycin. The target gene overexpression plasmids were transfected into 293 T cells, along with the helper plasmids psPAX2 and pMD2.G to generate lentivirus particles, which were subsequently used for the transduction of target cells. The lentiviral supernatant was added to the cell culture in the presence of 8 µg/mL polybrene, and the positive clones were acquired following puromycin selection. The BxPC3 and PANC1 cell lines transduced with an empty lentiviral vector were considered as the negative control (NC) group, and cells without lentivirus exposure were considered as the blank control (CON) group. The transduction efficiencies were determined by RT-qPCR and western blotting.

### RNA Extraction and RT-qPCR

Total RNA was isolated from the transfected BxPC3 and PANC1 cell lines. cDNA was synthesized from 2 µg of total RNA using reverse transcrip­tion, and RT-qPCR was conducted utilizing the following primers: (i) for MDR1, forward primer, 5′GATTGACAGCTACAGCACGGAAG 3′ and reverse primer, 5′CGGTCGGGTGGGATAGTTGAATA3′; and (ii) for MRP1, forward primer, 5′GACTTCGTTCTCAGGCACATCA3′ and reverse primer, 5′TCCTTCGGCAGACTCGTTGA3′. GAPDH was used as a reference gene.

### Western Blotting

Total protein was harvested after transduced cells were lysed in ice-cold RIPA buffer (Millipore, Temecula, CA, USA). Next, the cell lysates were separated using SDS-PAGE (Invitrogen, Carlsbad, CA, USA) and transferred to PVDF membranes. After being blocked with 5% skim milk, the membranes were incubated with the primary antibodies at 4°C overnight. After washing, the PVDF membranes were incubated with secondary antibodies for 2 h. The results were visualized by enhanced chemiluminescence (ECL) (Amersham, Chicago, IL, USA).

### Growth Inhibition Assays

The antitumor effects of GEM and GEM-HSA-NP on transduced pancreatic cancer cells were determined using the CCK8 assay. Transduced cells in the exponential growth phase were trypsinized and seeded into a 96-well plate (2,000 cells/well). After 24 h, the medium was replaced with 0.1 mL fresh medium dissolving 0.5 µg/mL GEM or GEM-HSA-NP containing equivalent GEM, and the cells were incubated at 37°C for a different time. 10 µL CCK8 was added to each well, followed by a 1 h incubation. The dye absorbance was read at 450 nm wavelength using an automatic spectrophotometer.

### Cell Cycle and Apoptosis Analyses

Transfected cells were incubated with 0.5 µg/mL GEM or GEM-HSA-NP containing equivalent GEM for 48 h. Flow cytometric cell cycle and apoptosis analyses were performed using an Accuri™ C6 Plus (Becton-Dickinson Biosciences, USA) flow cytometer. Cell cycle distribution was estimated using the appropriate software ModFit LT (Verity Software House, Inc., Topsham, ME, USA). The rate of apoptosis was determined using FlowJo software (BD Biosciences, San Jose, CA, USA).

### Detection of Intracellular ATP Levels

After treatment with 0.5 µg/mL GEM or GEM-HSA-NP containing equivalent GEM for 48 h, the intracellular ATP levels were determined using the luciferin–luciferase method by following the ATP assay kit protocol. Briefly, the cells were lysed in 200 µL of lysis buffer, centrifuged at 12,000 × g for 5 min, and the cell supernatant was collected. A 100 µL aliquot of the ATP detection working solution was added to each well of a black 96-well plate, and then the culture plate was incubated for 4 min at room temperature. 20 µL cell lysate samples were continuously added to each well, before immediately measuring their luminescence against the ATP standards supplied.

### Statistical Analyses

All the statistical analyses were conducted using GraphPad Prism 8 (Graph-Pad Software, San Diego, CA, USA). The Student’s *t*-test was used to analyze differences between two groups, and multiple groups were evaluated by One-Way analysis of variance (ANOVA) test. *P* < 0.05 was recognized as statistically significant.

## Results

### MDR1 and MRP1 Overexpression in Pancreatic Cancer Cell Lines

**Figure 1:**
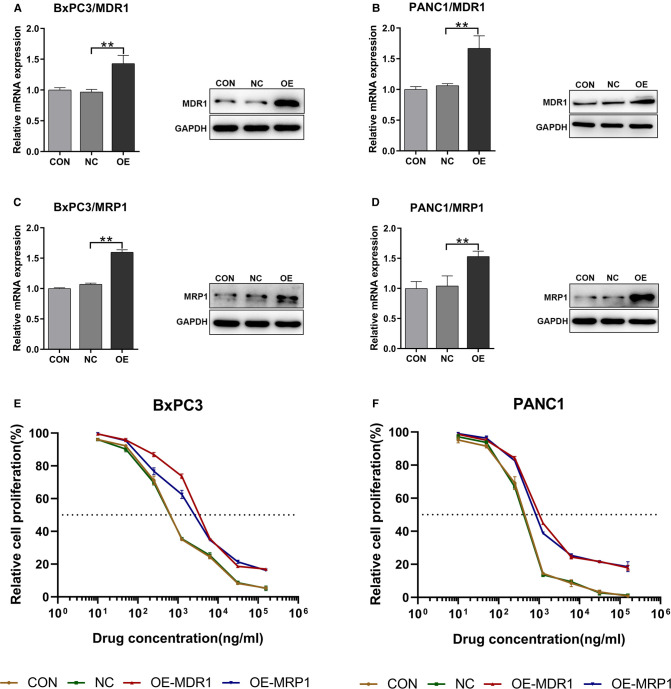
The upregulation of MDR1 and MRP1 in BxPC3 and PANC1 cell lines. Notes: RT-qPCR and western blotting were used to validate the transduction efficiency of BxPC3/MDR1 (**A**), PANC1/MDR1 (**B**), BxPC3/MRP1 (**C**), and PANC1/MRP1 (**D**) cell lines. Drug sensitivity before and after lentiviral transduction of BxPC3 (**E**) and PANC1 (**F**) cell lines was calculated after exposure to GEM for 48 h. **P < 0.01. Abbreviations: CON, cells without lentivirus treatment; NC, cells transduced with an empty vector; OE, cells transduced with the overexpression vector; OE-MDR1, cells transduced with the MDR1 overexpression vector; OE-MRP1, cells transduced with the MRP1 overexpression vector; GEM refers to gemcitabine.

To investigate the effect of GEM-HSA-NP on drug-resistant pancreatic cancer cells induced by high ABC transporter expression, we transduced the pancreatic cancer cell lines BxPC3 and PANC1 with *MDR1-* and *MRP1-*containing lentiviral vectors. We generated four pancreatic cancer cell types exhibiting ABC transporter overexpression: BxPC3/MDR1, PANC1/MDR1, BxPC3/MRP1, and PANC1/MRP1 (Figure 1), which we labelled as the OE group, referring to cells transduced with MDR1- or MRP1-containing lentiviral overexpression vectors. In addition, two groups of control cells lines were generated: the blank control (CON) group, which refers to cells that did not undergo lentiviral transduction; and the negative control (NC) group, which refers to cells that were transduced with an empty lentiviral vector. To qualify transduction efficiency, we determined the expression of *MDR1* and *MRP1* at the mRNA and protein levels by RT-qPCR and western blotting, respectively. Following the transduction of pancreatic cells with lentiviral *MDR1-* and *MRP1-*containing overexpressed vectors, the corresponding mRNA and protein levels within BxPC3/MDR1, PANC1/MDR1, BxPC3/MRP1, and PANC1/MRP1 cells were all significantly upregulated (*p* < 0.01, Figures 1A–D). At the same time, the expression levels of MDR1 and MRP1 in the NC group were mostly unchanged, compared to the CON group. To compare the variation in sensitivity to GEM experienced by BxPC3 and PANC1 cell lines after lentiviral transduction, IC_50_ values were measured by the CCK8 assay after cells were treated with GEM for 48 h. Experimental points presented in the drug sensitivity curves (Figures 1E,F) correspond to GEM concentrations of 10, 50, 250, 1,250, 6,250, 31,250, and 156,250 ng/mL. The IC_50_ and resistance index (RI) values are summarized in Table 1, which indicates that the IC_50_ values for the OE-MDR1 and OE-MRP1 groups were much higher than those for the NC group in both the BxPC3 and PANC1 cell lines.

**Table 1:** IC_50_ values (ng/mL) of pancreatic cancer cells.

	CON	NC	OE-MDR1	RI	OE-MRP1	RI
BxPC3	805.5	793.9	3947.0	4.97	2853.0	3.59
PANC1	413.5	393.3	1638.0	4.16	1421.0	3.61

*Notes: The IC_50_ values before and after lentiviral transduction of BxPC3 and PANC1 cell lines were calculated after exposure to GEM for 48 h.*

*Abbreviations: CON, cells without lentivirus treatment; NC, cells transduced with an empty vector; OE-MDR1, cells transduced with the MDR1 overexpression vector; OE-MRP1, cells transduced with the MRP1 overexpression vector; RI, resistance index; GEM refers to gemcitabine.*

### The Effect of GEM-HSA-NP on Growth Inhibition

**Figure 2:**
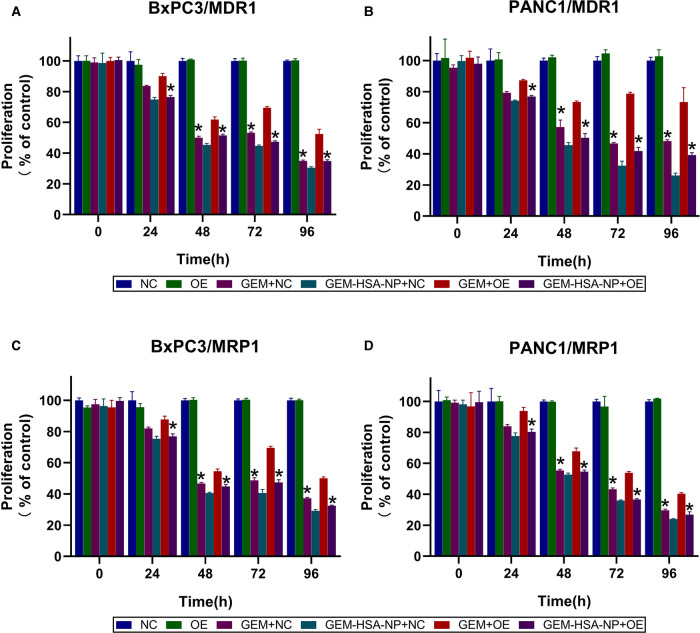
GEM-HSA-NP-mediated inhibition of MDR1- and MRP1-overexpressing BxPC3 and PANC1 cell proliferation. Notes: Cell viability was calculated at 0, 24, 48, 72, and 96 h after BxPC3/MDR1 (**A**), PANC1/MDR1 (**B**), BxPC3/MRP1 (**C**) and PANC1/MRP1 (**D**) cell lines were exposed to GEM or GEM-HSA-NP, and the extent of proliferation in each group is shown as bar charts. *: Significantly different from the GEM + OE group. Abbreviations: NC, cells transduced with an empty vector; OE, cells transduced with the overexpression vector; GEM refers to gemcitabine; GEM-HSA-NP refers to gemcitabine-loaded human serum albumin nanoparticle.

CCK8 assays were utilized to evaluate the capacity of GEM-HSA-NP to inhibit the proliferation of transduced BxPC3 and PANC1 cell lines. Cell viability was determined at 0, 24, 48, 72, and 96 h after the transduced cells were treated with various drugs. As is shown in Figures 2A,B, the growth rate of cells belonging to the OE group was similar to that of the NC group, indicating that the overexpression of MDR1 did not affect cell proliferation. In addition, on the whole, both GEM and GEM-HSA-NP inhibited cell proliferation in a time-dependent manner, and higher levels of cytotoxicity were observed at longer incubation times.

GEM and GEM-HSA-NP exerted similar inhibitory effects on the proliferation of cells belonging to the NC group. However, the inhibitory effect of GEM on the proliferation of OE cells was much weaker than that of GEM-HSA-NP (*p *< 0.05, Figures 2A,B), demonstrating that pancreatic cancer cells overexpressing MDR1 showed significant resistance to GEM. The same experiments were repeated on the MRP1-overexpressing pancreatic cancer cell lines BxPC3/MRP1 and PANC1/MRP1 (Figures 2C,D), with similar results.

### The Effect of GEM-HSA-NP on Cell Cycle Arrest

**Figure 3:**
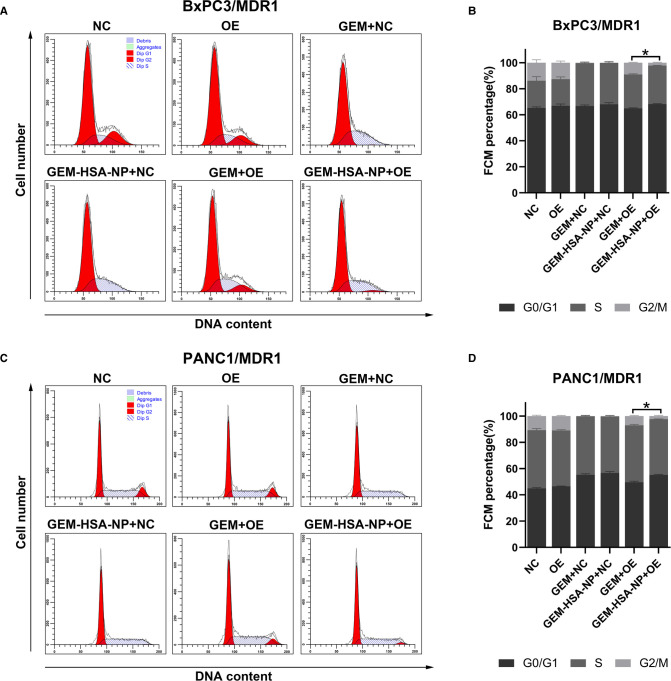
GEM-HSA-NP-mediated cells cycle arrest of MDR1-overexpressing BxPC3 and PANC1 cells. Notes: The proportions of cells in each phase of the cell cycle were evaluated by flow cytometry after the BxPC3/MDR1 (**A**) and PANC1/MDR1 (**C**) cell lines were exposed to GEM or GEM-HSA-NP for 48 h. Percentage of cells in each BxPC3/MDR1 (**B**) and PANC1/MDR1 (**D**) group at each phase of the cell cycle is shown as bar charts. *: The percentage of cells in the G2/M phase is significantly different. Abbreviations: NC, cells transduced with an empty vector; OE, cells transduced with the overexpression vector; GEM refers to gemcitabine; GEM-HSA-NP refers to gemcitabine-loaded human serum albumin nanoparticle.

**Figure 4:**
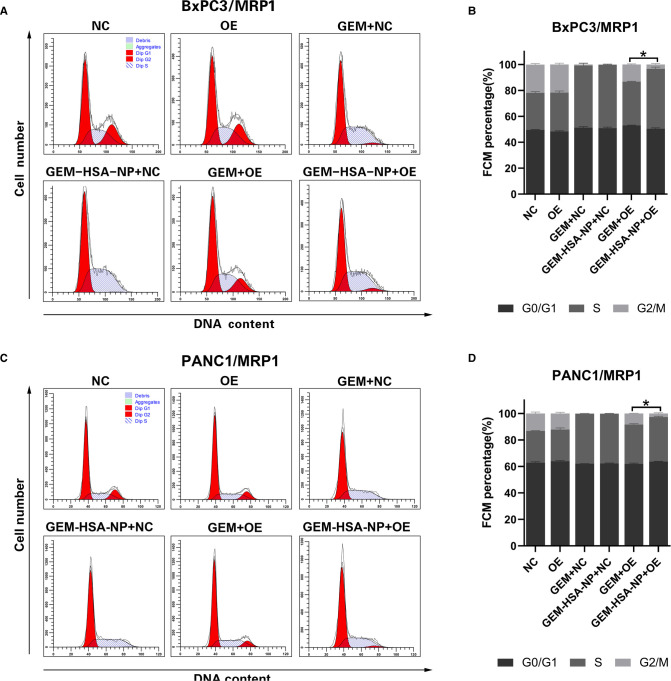
GEM-HSA-NP-mediated cells cycle arrest of MRP1-overexpressing BxPC3 and PANC1 cells. Notes: The proportions of cells in each phase of the cell cycle were evaluated by flow cytometry after BxPC3/MRP1 (**A**) and PANC1/MRP1 (**C**) cell lines were exposed to GEM or GEM-HSA-NP for 48 h. Percentage of cells in each phase of the cell cycle in each group of BxPC3/MRP1 (**B**) and PANC1/MRP1 (**D**) cell lines is shown as bar charts. *: The percentage of cells in the G2/M phase is significantly different. Abbreviations: NC, cells transduced with an empty vector; OE, cells transduced with the overexpression vector; GEM refers to gemcitabine; GEM-HSA-NP refers to gemcitabine-loaded human serum albumin nanoparticle.

The cycling of cells was investigated to examine the ability of GEM-HSA-NP to cause cell cycle arrest in MDR1- or MRP1-overexpressing GEM-resistant pancreatic cancer cells. The distribution of pancreatic cancer cells at different cell cycle phases after being treated with GEM and GEM-HSA-NP for 48 h is presented in Figures 3, 4. We began by analyzing the cell cycle of MDR1-overexpressing pancreatic cancer cell lines in the presence of GEM and GEM-HSA-NP. When NC group cells were exposed to GEM or GEM-HSA-NP, a larger number of cells accumulated in the S phase (BxPC3/MDR1, Figure 3A) or in the G0/G1 phase (PANC1/MDR1, Figure 3C) while a smaller proportion of cells were observed in the G2/M phase, indicating that the cell cycle of NC group cells was effectively arrested by GEM and GEM-HSA-NP treatment. In contrast, the cell cycle of the GEM-treated OE group cells was restored to a relatively normal level, similar to that of the untreated OE groups, which demonstrates the resistance of MDR1-overexpressing cancer cells to GEM. However, the cell cycle of OE group cells treated with GEM-HSA-NP was still arrested at a relatively high level. Quantitative analysis of the cell cycle distribution illustrated that the percentage of cells in the G2/M phase of the GEM-HSA-NP + OE group was significantly reduced in comparison with the GEM + OE group (9.03 ± 0.34% vs. 2.20 ± 0.35% in BxPC3/MDR1, *p* < 0.05, Figure 3B; 7.15 ± 0.43% vs. 2.46 ± 0.55% in PANC1/MDR1, *p* < 0.05, Figure 3D). We subsequently evaluated the cell cycles dynamics of MRP1-overexpressing pancreatic cancer cell lines. The results showed that the cell cycles of BxPC3/MRP1 (Figure 4A) and PANC1/MRP1 (Figure 4C) cells were both arrested at the S phase. Again, quantitative analysis of cell cycle distribution demonstrated that the percentage of cells in the G2/M phase of the GEM-HSA-NP + OE group was significantly lower than the GEM + OE group (13.36 ± 0.48% vs. 3.46 ± 0.73% in BxPC3/MRP1, *p* < 0.05, Figure 4B; 8.43 ± 0.17% vs. 2.74 ± 0.54% in PANC1/MRP1, *p* < 0.05, Figure 4D).

### The Effect of GEM-HSA-NP On Cell Apoptosis

**Figure 5:**
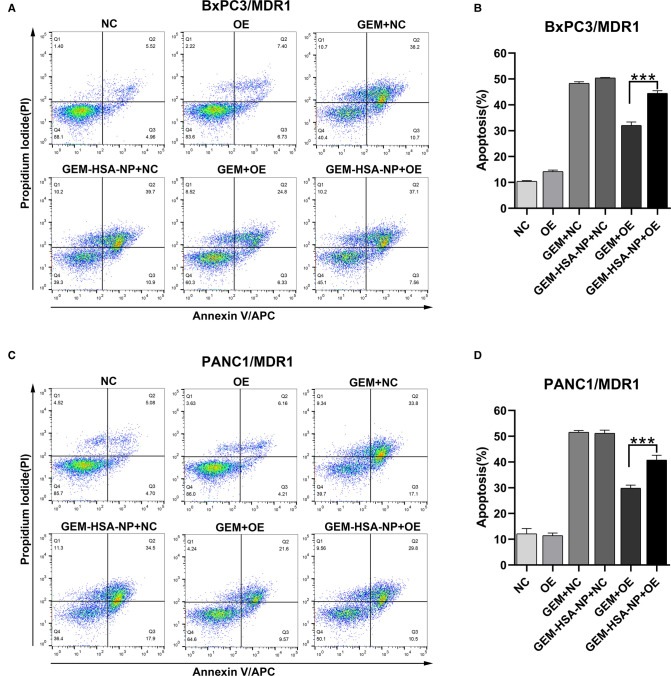
GEM-HSA-NP-mediated apoptosis of MDR1-overexpressing BxPC3 and PANC1 cells. Notes: The extent of apoptosis was evaluated by flow cytometry after BxPC3/MDR1 (**A**) and PANC1/MDR1 (**C**) cell lines were exposed to GEM or GEM-HSA-NP for 48 h. Apoptosis rates in each group of BxPC3/MDR1 (**B**) and PANC1/MDR1 (**D**) cell lines are shown as bar charts. ***P < 0.001. Abbreviations: NC, cells transduced with an empty vector; OE, cells transduced with the overexpression vector; GEM refers to gemcitabine; GEM-HSA-NP refers to gemcitabine-loaded human serum albumin nanoparticle.

**Figure 6:**
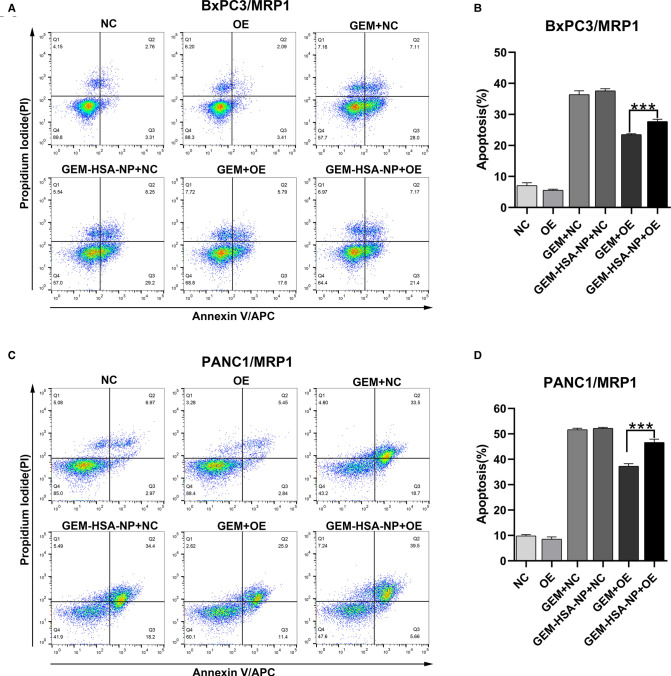
GEM-HSA-NP-mediated apoptosis of MRP1-overexpressing BxPC3 and PANC1 cells. Notes: The extent of apoptosis was evaluated by flow cytometry after BxPC3/MRP1 (**A**) and PANC1/MRP1 (**C**) cell lines were exposed to GEM or GEM-HSA-NP for 48 h. Apoptosis rates in each group of BxPC3/MRP1 (**B**) and PANC1/MRP1 (**D**) cells are shown as bar charts. ***P < 0.001. Abbreviations: NC, cells transduced with an empty vector; OE, cells transduced with the overexpression vector; GEM refers to gemcitabine; GEM-HSA-NP refers to gemcitabine-loaded human serum albumin nanoparticle.

To explore the differences in the abilities of GEM and GEM-HSA-NP to induce apoptosis in GEM-resistant pancreatic cells lines, we performed apoptosis assays using the Annexin V-APC/PI apoptosis detection kit after lentivirally-transduced cells were treated with GEM or GEM-HSA-NP for 48 h (Figures 5, 6). GEM and GEM-HSA-NP were both able to increase apoptosis rates of BxPC3/MDR1 (Figure 5A), PANC1/MDR1 (Figure 5C), BxPC3/MRP1 (Figure 6A), and PANC1/MRP1 (Figure 6C) cell lines, within the NC group. However, the pro-apoptotic effect of GEM was markedly reduced when applied to cells within the OE group, confirming that the overexpression of ABC transporters results in GEM resistance. In contrast, the apoptosis rate of cells in the OE group caused by GEM-HSA-NP treatment remained at a relatively high level. Quantitative analysis of cell apoptosis (including both early-stage and late-stage apoptosis) demonstrated that the apoptosis rate in the GEM-HSA-NP + OE group was significantly higher than that in the GEM + OE group (44.50 ± 0.99% vs. 32.07 ± 1.29% in BxPC3/MDR1, *p* < 0.001, Figure 5B; 40.80 ± 1.80% vs. 29.89 ± 1.14% in PANC1/MDR1, *p* < 0.001, Figure 5D; 27.73 ± 0.73% vs. 23.50 ± 0.29% in BxPC3/MRP1, *p* < 0.001, Figure 6B; 46.62 ± 1.31% vs. 37.30 ± 1.00% in PANC1/MRP1, *p* < 0.001, Figure 6D).

### The Effect of GEM-HSA-NP on Intracellular ATP Levels

**Figure 7:**
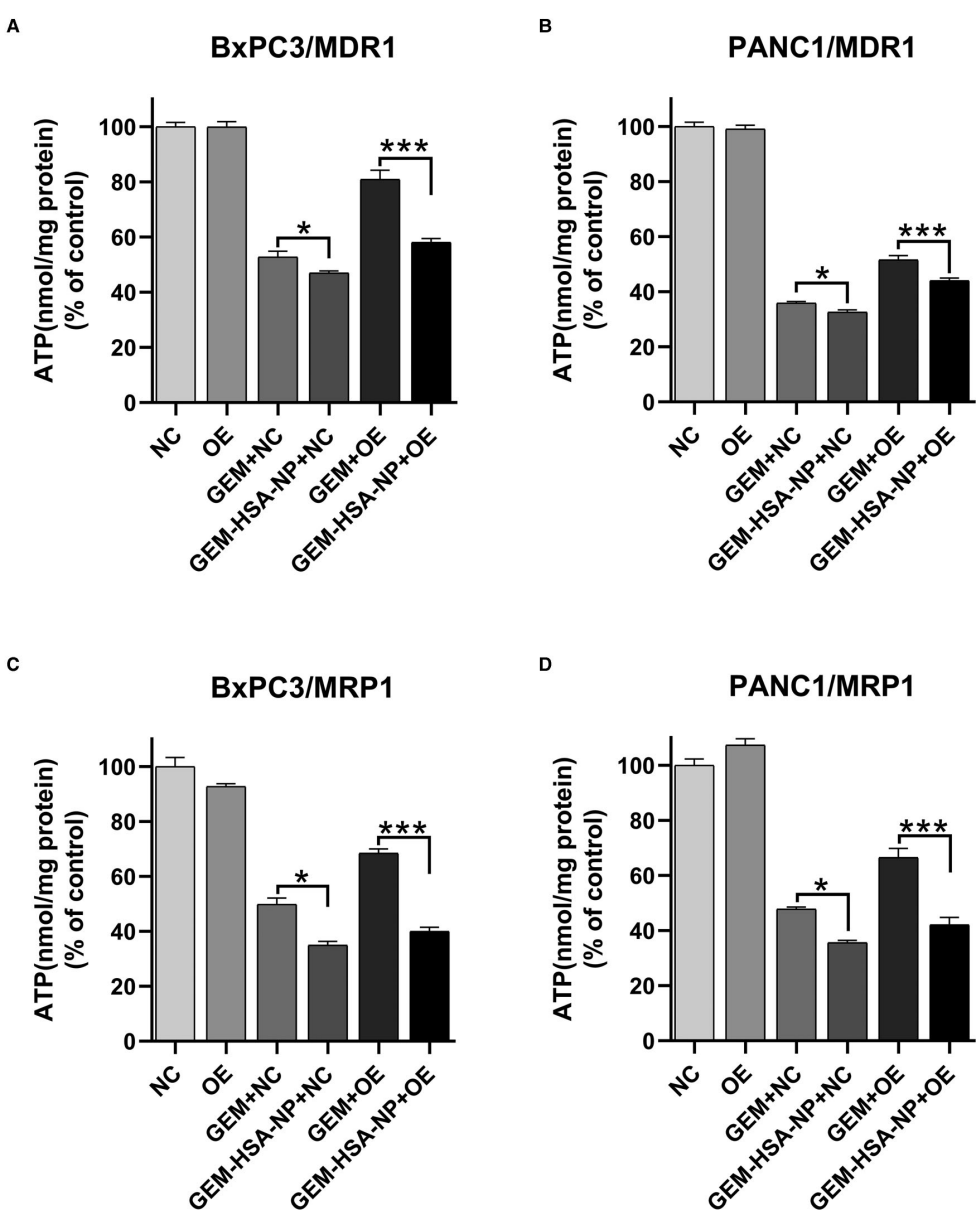
The effect of GEM-HSA-NP on the intracellular ATP levels of MDR1- and MRP1-overexpressing pancreatic cancer cell lines. Notes: Intracellular ATP levels were detected after BxPC3/MDR1 (**A**), PANC1/MDR1 (**B**), BxPC3/MRP1 (**C**), and PANC1/MRP1 (**D**) cell lines were exposed to GEM or GEM-HSA-NP for 48 h. *P < 0.05, ***p < 0.001. Abbreviations: NC, cells transduced with an empty vector; OE, cells transduced with the overexpression vector; GEM refers to gemcitabine; GEM-HSA-NP refers to gemcitabine-loaded human serum albumin nanoparticle.

Intracellular ATP levels were measured after the four cell lines (BxPC3/MDR1, PANC1/MDR1, BxPC3/MRP1, and PANC1/MRP1) were exposed to GEM or GEM-HSA-NP for 48 h. As indicated in Figure 7, after the cells were treated with either GEM or GEM-HSA-NP, the intracellular ATP levels of each cell group decreased by various degrees, reflecting the cytotoxicity of GEM and GEM-HSA-NP. For the NC group cells, both GEM and GEM-HSA-NP treatment effectively reduced intracellular ATP levels, notably, the ATP level in the GEM-HSA-NP + NC group was significantly lower than the GEM + NC group (46.98 ± 0.75% vs. 52.79 ± 2.09 in BxPC3/MDR1, *p* < 0.05, Figure 7A; 32.58 ± 0.87% vs. 35.93 ± 0.49% in PANC1/MDR1, *p* < 0.05, Figure 7B; 34.94 ± 1.37% vs. 49.80 ± 2.31% in BxPC3/MRP1, *p* < 0.05, Figure 7C; 35.63 ± 0.81% vs. 47.82 ± 0.75% in PANC1/MRP1, *p* < 0.05, Figure 7D). For the OE group cells, the ATP level of the GEM-HSA-NP-treated group was significantly reduced compared with the GEM-treated group (58.04 ± 1.36% vs. 80.88 ± 3.36 in BxPC3/MDR1, *p* < 0.001; 44.08 ± 0.93% vs. 51.57 ± 1.57% in PANC1/MDR1, *p* < 0.001; 40.04 ± 1.48% vs. 68.42 ± 1.58% in BxPC3/MRP1, *p* < 0.001; 42.17 ± 2.66% vs. 66.47 ± 3.33% in PANC1/MRP1, *p* < 0.001).

## Discussion

As a highly malignant cancer, pancreatic cancer is an increasing threat to human beings, with its treatment facing many challenges. Drug resistance has greatly reduced the effectiveness of existing chemotherapy regimens, which is a key problem in urgent need for effective solutions (32). Since nab-paclitaxel was recommended as the first-line treatment option for advanced pancreatic cancer, the synthesis of albumin nanoparticles has attracted rising attention from reserchers. It was reported that curcumin-loaded albumin nanoparticle have been prepared for intravenous administration using nano-binding technology (33). In our previous research, we used human serum albumin as a carrier of gemcitabine, synthesized gemcitabine albumin nanoparticle by nano-binding technology, and verified its improved antitumor effect on pancreatic cancer (29). Here, we further explored its efficacy in overcoming drug resistance induced by MDR1 and MRP1 overexpression in pancreatic cancer.

The conventional approach to construct drug-resistant cells is increasing drug exposure concentration gradually, however, the mechanisms of chemoresistance discovered by studying drug-induced drug-resistant cell lines are typically multi-factorial and complex, moreover, the drug resistance exhibited by these cells lines is often unstable (34, 35). Due to the presence of multiple confounding factors and unstable drug-resistant phenotypes, drug-resistant cell lines induced by a stepwise increase in the concentration of chemotherapeutic drugs are not ideal models for our study. To evaluate the antitumor effect of GEM-HSA-NP on pancreatic cancer cells expressing high levels of ABC transporters more accurately, we performed lentiviral transduction to construct single-factorial drug-resistant pancreatic cancer cells lines. The higher MDR1 and MRP1 expression levels and the apparent resistance of the transduced cells to GEM, as illustrated by the experimental data, confirmed that the MDR1 and MRP1 single-factor drug-resistant cell lines, BxPC3/MDR1, PANC1/MDR1, BxPC3/MRP1, and PANC1/MRP1, were successfully established. Furthermore, since their drug-resistant phenotype was conferred by *MDR1* and *MRP1* gene overexpression alone and in the absence of any other factors, the established cell lines may possess higher specificity than drug-induced drug-resistant cells. Thus, our lentivirally-transduced cell lines circumnavigate the complex multi-parameter resistance mechanisms found in conventional cellular models, which may provide more accurate and intuitive data.

The CCK8 assay demonstrated that compared to free GEM, the proliferation-inhibiting effect of GEM-HSA-NP on MDR1- and MRP1-overexpressing pancreatic cancer cells remained potent. Cell cycle regulation plays a vital role in the modulation of cell growth (36, 37). GEM is a DNA inhibitor that can arrest the cell cycle in the G0/G1 or S phases (38, 39). Experimental results showed that when the cycle arrest effect of GEM on drug-resistant pancreatic cancer cells was markedly reduced, GEM-HSA-NP still exerted effective cycle arrest ability. Apoptosis induction also acts as an essential mechanism for regulating cell proliferation (40, 41). For drug-resistant cells, the apoptosis-inducing effect of GEM was significantly inhibited, however, this effect of GEM-HSA-NP retained at a relatively high level. The proliferation inhibition, cell cycle arrest, and apoptosis induction assays described above all conducted in these four drug-resistant pancreatic cancer cells, including BxPC3/MDR1, PANC1/MDR1, BxPC3/MRP1, and PANC1/MRP1, and these data of these four pancreatic cancer cell lines displayed basically consistent trends, which collectively indicated that GEM-HSA-NP could effectively inhibit proliferation, arrest cell cycle, and induce apoptosis of MDR1- and MRP1-overexpressing pancreatic cancer cells, thus reversing GEM resistance.

The overexpression of ABC transporters in cancer cells is regarded as the main reason for multidrug resistance (42). ABC transporters have been shown to pump substrates, including GEM, out of tumor cells via an ATP-dependent mechanism, reducing effective drug concentration and thereby weakening the antitumor effect of chemotherapeutic drugs (43). The function of ABC transporters is highly dependent on the intracellular levels of ATP, which is hydrolyzed to pump substrates out of the cell (44). Notably, the degradation of exogenous proteins after being absorbed into the cell also requires ATP (31). Therefore, to explore the possible mechanism employed by GEM-HSA-NP to overcome GEM resistance preliminarily, we measured intracellular ATP levels, and observed how they were affected by GEM-HSA-NP.

As the most important energy molecule, ATP plays a pivotal role in various physiological and pathological cellular processes. When cells are apoptotic, necrotic, or subjected to cytotoxicity, intracellular ATP levels usually decrease (45). In our study, the reduction in intracellular ATP levels was mainly due to two factors: the cytotoxic effect of GEM and the degradation of albumin. The ATP level in the GEM + OE group was markedly higher than the GEM + NC group, indicating that the cytotoxicity was reduced because a portion of intracellular GEM in the cell was transported outside the cell by the overexpressed ABC transporters, lowering intracellular GEM concentration and consequently raising the intracellular ATP level. By comparing the GEM + NC and GEM-HSA-NP + NC groups, the ATP level of the latter was significantly lower than the former, which confirmed that the degradation of albumin, a component of GEM-HSA-NP, could indeed lead to ATP depletion following its absorption by cells. The comparison of the GEM + OE and GEM-HSA-NP + OE groups revealed that the ATP level of the GEM-HSA-NP + OE group was significantly lower than that of the GEM + OE group, implying a connection between the anti-drug resistance effect of GEM-HSA-NP and intracellular ATP levels.

Based on the above results and given that the function of ABC transporters is highly dependent on intracellular ATP levels, we speculated that in the GEM-HSA-NP + OE group, the ATP consumed by albumin degradation might lead to a relatively insufficient supply of ATP for optimal ABC transporter function. Consequently, the efflux activity of ABC transporters was reduced, resulting in the retention of more cytotoxic GEM in the cell. Thus, we postulated that the competitive consumption of ATP by albumin degradation following GEM-HSA-NP administration might serve as a factor in the reversal of multidrug resistance by GEM-HSA-NP, and seemingly, loading more HSA in GEM-HSA-NP might be helpful to strengthen this effect. Some previous researches in human lung cancer cells illustrated that intracellular ATP depletion was related to modulation of multidrug resistance (46, 47). Our research results are also in accordance with this hypothesis. However, since we merely measured the variation in intracellular ATP levels, it remains to be determined to what extent the ability of GEM-HSA-NP to reverse GEM resistance derives from the depletion of ATP by albumin degradation, or whether GEM-HSA-NP employs other mechanisms that affect the function of ABC transporters. The limitation of our current study is that all the experiments were conducted at the in vitro level, therefore, the therapeutic evaluations and superior antitumor effect of GEM-HSA-NP on MDR1- and MRP1-overexpressing pancreatic cancers needs to be further elucidated in vivo in our future work.

In conclusion, ABC transporters are involved in the efflux of GEM, and the high expression of MDR1 and MRP1 can result in GEM resistance. GEM-HSA-NP can effectively overcome GEM resistance induced by MDR1 and MRP1 overexpression in pancreatic cancer cells. Furthermore, competitive ATP depletion as a result of the degradation of GEM-HSA-NP-derived albumin might be a contributing factor. Further in-depth studies are needed to explore the underlying mechanisms involved in the reversal of drug-resistance by GEM-HSA-NP and assess its clinical value in the treatment of cancer.

## Data Availability

The data analysed in the current study are available from the corresponding author on reasonable request.
